# Autistic symptoms in Greig cephalopolysyndactyly syndrome: a family case report

**DOI:** 10.1186/s13256-019-2043-6

**Published:** 2019-04-23

**Authors:** Martina Siracusano, Assia Riccioni, Antonia Baratta, Maurizia Baldi, Paolo Curatolo, Luigi Mazzone

**Affiliations:** 1 Department of Biomedicine and Prevention, University of Rome Tor Vergata, Rome, Italy; 2 Department of Biotechnological and Applied Clinical Sciences, Experimental Medicine-Neuroscience, University of L’Aquila, L’Aquila, Italy; 3 System Medicine Department, Child Neurology and Psychiatry Unit, University of Rome Tor Vergata, Rome, Italy; 4 S.C. Human Genetic Laboratory, Ospedali Galliera, Genoa, Italy

**Keywords:** Greig cephalopolysyndactyly syndrome, Autism, Neuropsychological phenotype, Development, Intellectual disability, Comorbidity

## Abstract

**Background:**

Greig cephalopolysyndactyly syndrome is a rare multiple congenital anomaly syndrome characterized by the triad of polysyndactyly (preaxial or mixed preaxial and postaxial), macrocephaly, and ocular hypertelorism. Little is known about the neuropsychological phenotype and the developmental features of this syndrome.

**Case presentation:**

We describe the clinical features of a 7-year-old Italian white boy affected by Greig cephalopolysyndactyly syndrome in comorbidity with autism spectrum disorder and the case of his 45-year-old white father, carrying the same point deletion (c.3677del) in the *GLI3* gene and showing subclinical autistic symptoms. We performed a neuropsychiatric assessment of cognitive, adaptive, socio-communicative, and behavioral skills of the child. Concurrently, the father underwent his first psychiatric evaluation of cognitive skills and autistic symptoms.

**Conclusions:**

We report the first clinical description of an association between autistic symptoms and Greig cephalopolysyndactyly syndrome in two members of the same family with the same genetic point deletion. Further research is required in order to draw an accurate conclusion regarding the association between Greig cephalopolysyndactyly syndrome and autism.

## Background

Greig cephalopolysyndactyly syndrome (GCPS), Online Mendelian Inheritance in Man (OMIM) 175700, is a rare (estimated incidence 1–9/1,000,000) multiple congenital anomaly syndrome inherited in an autosomal dominant pattern [1]. Loss of function mutations on chromosome 7p14.1 in the transcription factor gene *GLI3* determine GCPS, which is allelic to Pallister–Hall syndrome (OMIM 146510) [2]. The *GLI3* gene regulates cell growth, proliferation, and specialization (limbs, brain) and its expression is essential during early stages of development [3].

This syndrome is clinically characterized by the typical triad of polysyndactyly (preaxial or mixed preaxial and postaxial), macrocephaly, and ocular hypertelorism [4]. However, a wide spectrum of phenotypic variation has been described with a high variability of major clinical findings in terms of the expressivity and severity of the disease [1, 4].

Little is known about the neuropsychological phenotype and the developmental features of this syndrome. Intellectual disability and developmental delay are not common manifestations of the classical syndrome [1]. Up to now and to the best of our knowledge, there is only one study in the literature reporting mild-severe developmental delay in 11–20% of patients with GCPS [5]. Intellectual disability and developmental delay together with hydrocephalus and seizures are included in a more severe clinical phenotype [4, 6, 7] associated with a variant disorder characterized by a worse prognosis: the Greig cephalopolysyndactyly-contiguous gene syndrome (GCPS-CGS), caused by mutations larger than 1 Mb in the *GLI3* gene [8, 9].

To the best of our knowledge, autistic symptoms have not been previously described in association with GCPS neither in the classical syndrome nor in its variant form (GCPS-CGS).We describe the case of a 7-year-old boy affected by GCPS and autism spectrum disorder (ASD) and the case of his 45-year-old father, also affected by the syndrome and with subclinical autistic symptoms.

## Case presentation

### Child

A 7-year-old Italian white boy affected by GCPS and ASD was referred to our Child Psychiatry Unit for a neuropsychiatric assessment.

The child, born of non-consanguineous white parents, was born at 40 weeks of gestation by vaginal delivery. A previous spontaneous miscarriage was reported. His birth weight was 3070 g (15–50th centile), length 49 cm (15–50th centile), head circumference 34 cm (15–50th centile), and APGAR Index 9–10. He was born with postaxial polysyndactyly of his hands (right hand had two extra fingers, partial syndactyly of finger 5–6; left hand had one extra finger) and of his right foot (one extra toe), surgically corrected at 6 months of age. In the early perinatal period, due to the observed dysmorphic features, the child underwent brain ultrasound (referred as normal) and genetic counseling without specific indication for subsequent genetic screening.

Motor developmental milestones were normally achieved. A history of language delay was reported: first words at 18 months with a following regression of the verbal development. At around 30 months of age, restricted and repetitive behaviors (RRBs), social withdrawal, lack of pretending game together with poor communicative skills were the main parental worries. Based on these clinical features, at 3 years of age a diagnosis of ASD was made and for this reason he started applied behavior analysis (ABA) behavioral therapy (12 hours per week).

**Fig. 1:**
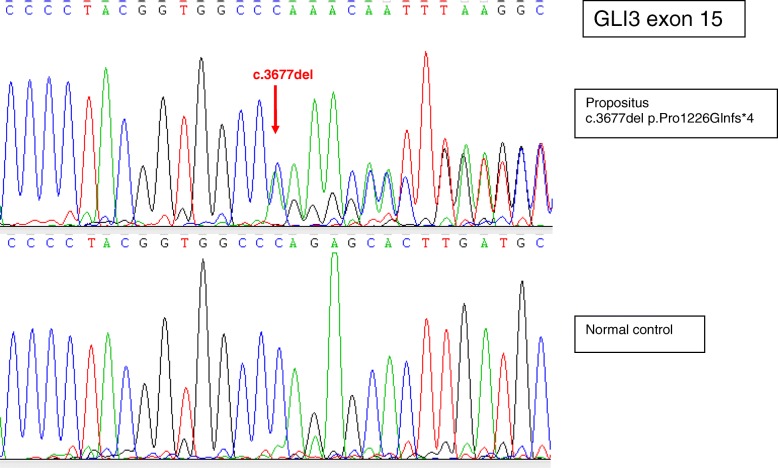
Sequence chromatogram. Sequence chromatogram showing the point mutation (c.3677del in the *GLI3* gene) compared to a normal control

The diagnosis of GCPS was clinically suspected in both the child and his father respectively at 3 and 42 years of age, and later molecularly confirmed through direct sequencing and multiplex ligation-dependent probe amplification (MLPA): “heterozygous for the single nucleotide deletion c.3677del, point mutation paternally transmitted, not previously described, localized in gene’s region associated with GCPS, resulting in a truncated GLI3 protein caused by the frameshift mutation and the insertion of a premature stop codon (Pro1226Glnfs4)” (see Fig. 1 for the chromatogram).

Asymmetry of the ventricular supratentorial system (right major representation) and lateral deviation of a septum pellucidum were present on brain magnetic resonance imaging performed at 6 years of age; whereas a routine and sleep-induced electroencephalogram recorded diffused paroxysmal abnormal activity during the falling asleep phase and decreasing during sleep.

No history of clinical seizures was reported. Sleep/wake cycle was regular. Food selectivity was reported since 30 months of age.

On our clinical examination at 7 years and 5 months of age, he weighed 26 kg (50–85th centile), his height was 126 cm (50–85th centile), and his head circumference around was 54.3 cm (98th centile); frontal bossing, a prominent forehead, hypertelorism, a flat nasal bridge, and low-set ears were present. A neurological examination showed normal cranial nerves, and regular muscular tropism and tone. No sensory or autonomic involvement was observed. Deep tendon reflexes of superior and lower limbs were present and normal. The Finger-to-Nose test, performed with open eyes, due to lack of collaboration, showed mild hesitation. A widespread ligament lassitude and a mild deficiency of superior limbs’ strength were observed.

We performed a neuropsychiatric assessment of cognitive, adaptive, socio-communicative, and behavioral skills through standardized tools (see Table 1); in detail, the Leiter International Performance Scale, Third Edition (Leiter-3) [10] and the Coloured Progressive Matrices (CPM) [11] were administered revealing a non-verbal intellectual quotient (IQ) of 71 and inclusion between 10 and 25th centile (range 75–85).

**Table 1:** Child’s neuropsychological findings

CPM	10–25th centile (IQ range 75–85)
LEITER-3	IQ 71
ABAS-II	Composite score: General Adaptive 56; Conceptual 63; Social 66; Practical 34
ADOS-2 (Module 1)	SA 16; RRB 7; Total 23; CSS 8
SRS	T score: Total 69; Social Awareness 67; Social Cognition 71; Social Communication 70; Social Motivation 80; Autistic Mannerism 70
CBCL 6–18 years	T score: Affective Problems = 60; Anxiety = 69; Somatic = 50; Attention Deficit/Hyperactivity = 60; Oppositional = 50; Conduct = 50

*ABAS-II* Adaptive Behavior Assessment System, Second Edition, *ADOS-2* Autism Diagnostic Observation Schedule, Second Edition, *CBCL* Child Behavior Checklist, *CPM* Coloured Progressive Matrices, *CSS* Calibrated Severity Score, *IQ* intellectual quotient, *LEITER-3* Leiter International Performance Scale, Third Edition, *RRB* restricted and repetitive behavior*, SA* social affect, *SRS* Social Responsiveness Scale

The Adaptive Behavior Assessment System, Second Edition (ABAS-II) [12], a questionnaire filled in by the caregivers evaluating ten adaptive areas organized in four main domains (General Adaptive, Conceptual, Social, Practical), showed adaptive skills below the average in all the fields evaluated (see Table 1).

The previous diagnosis of ASD was confirmed through clinical observation and the administration of the Autism Diagnostic Observation Schedule, Second Edition (ADOS-2), which is the gold standard instrument for the evaluation and diagnosis of autism [13]. We performed Module 1, which is suitable for children beyond 30 months of age with a verbal language composed of single words. The diagnostic algorithm is organized in two main areas: social affect (SA) and RRB. The total score obtained (SA 16 + RRB 7 = 23) exceeded the cut-off (16) for the diagnosis of autism. Finally, a moderate level of ASD symptom severity was measured through the Calibrated Severity Score (ADOS-CSS).

A Social Responsiveness Scale (SRS) [14], a questionnaire filled in by the parents, showed a moderate deficiency of the child’s social relationship, which compromised his general functioning.

Finally, no significant problematic behavior emerged from the caregiver report, Child Behavior Checklist (CBCL) [15], except for a borderline score in the area investigating anxiety problems (see Table 1).

### Father

The father’s clinical and molecular diagnosis of GCPS was made together with his son’s genetic consultation. Both carried the same single nucleotide deletion in the *GLI3* gene (c.3677del). Until 42 years of age he underwent no genetic examination.

He was born of Italian non-consanguineous white parents with postaxial polydactyly of the hands and of the right foot and congenital clubfoot, which were surgically operated on after birth. No genetic counseling and screening were performed in the perinatal and postnatal period. Developmental milestones were referred as normal. No academic difficulties were reported and he graduated with success. No family history of neuropsychiatric diseases emerged.

Concurrently with the child’s evaluation, we performed a neuropsychological assessment of the 45-year-old father. Until our clinical examination, he had never undergone a psychiatric evaluation. In particular, a cognitive assessment and a specific evaluation of autistic symptoms were performed (see Table 2). His non-verbal IQ, measured by Standard Progressive Matrices (SPM) [16], turned out to be above average (IQ 128). Autistic symptoms were measured with the ADOS-2 [13]. We performed Module 4, which is suitable for adults with fluent speech. The diagnostic algorithm was composed of two main domains: Communication domain (C domain) and Social Relationship domain (SR domain); the algorithm revealed a total score of 5 (C domain 2 + SR domain 3) which does not exceed the general cut-off for the “spectrum” (7) or for “autism” (10). The partial score of the C domain, however, reached the cut-off for the “spectrum” (2).

**Table 2:** Father’s neuropsychological findings

SPM	IQ 128
ADOS-2 (Module 4)	Communication domain 2; Social Relationship domain 3; Total 5; Imagination 0; RRB 0

*ADOS-2* Autism Diagnostic Observation Schedule, Second Edition, *IQ* intellectual quotient, *RRB* restricted and repetitive behavior, *SPM* Standard Progressive Matrices

## Discussion

GCPS is a rare autosomal dominant condition characterized by high variability of the phenotype. However, a minor intrafamilial severity is generally described among multiplex families (more than one member affected) [1]. A good prognosis of the patient is frequently reported and the risk of intellectual disability is usually related to the size of the deletions in the *GLI3* gene. The involvement of contiguous genes (GCPS-CGS) is associated with a worse prognosis and a major risk of cognitive impairment.

We reported the first description of autistic symptoms within individuals affected by GCPS. In particular, we described the case of two members of the same family, father and son, both carrying the same point deletion (c.3677del) in the *GLI3* gene and showing autistic symptoms, but at a different level. As a matter of fact, the child presented a definite diagnosis of ASD characterized by a moderate level of severity in association with cognitive and language impairment.

On the other hand, sub-threshold autistic symptoms emerged from the father’s clinical interview and the administration of the ADOS-2 in the C domain, with results highlighting a mild impairment. During the clinical observation, a prosodic deficit with low volume of the voice and a lacking of facial expressions directed to the examiner were observed; few voluntary emotional, instrumental gestures were associated with verbal language. Moreover, he described himself as a shy person with difficulties in maintaining eye contact during conversation. These communication difficulties have partially impaired his social functioning especially during adolescence.

Furthermore, although they both carried the same point mutation (smaller than 1 Mb), as emerged from the cognitive and adaptive evaluation, the child was affected by a mild intellectual disability, whereas the father’s IQ was above average. These differences in the severity of the neuropsychological phenotype (cognitive and socio-communicative) within the same family are in contrast with the literature that refers to less intrafamilial variability [1].

## Conclusions

This clinical case is the first description of an association between autistic symptoms and GCPS in two members of the same family with the same genetic point deletion. Autistic symptoms, either at a clinical or subclinical level, should be considered and investigated within the GCPS population in order to perform an early diagnosis and to begin the early intervention required. Therefore neuropsychiatric assessment is recommended in early stages of development.

Further descriptions of the cognitive and socio-communicative phenotype of the affected patients will enable us to better delineate a specific neuropsychological profile and developmental trajectory of the syndrome.

## Abbreviations

ABA: Applied behavior analysis
ABAS-II: Adaptive Behavior Assessment System, Second Edition
ADOS-2: Autism Diagnostic Observation Schedule, Second Edition
ASD: Autism spectrum disorder
CBCL: Child Behavior Checklist
C domain: Communication domain
CPM: Coloured Progressive Matrices
CSS: Calibrated Severity Score
GCPS: Greig cephalopolysyndactyly syndrome
GCPS-CGS: Greig cephalopolysyndactyly-contiguous gene syndrome
IQ: Intellectual quotient
MLPA: Multiplex ligation-dependent probe amplification
OMIM: Online Mendelian Inheritance in Man
RRB: Restricted and repetitive behavior
SA: Social affect
SPM: Standard Progressive Matrices
SR domain: Social Relationship domain
SRS: Social Responsiveness Scale
